# A systematic review of virtual elective programmes for medical students

**DOI:** 10.1111/tct.13841

**Published:** 2024-11-06

**Authors:** Luke McCarron, Nihal Sogandji, Luke Coakham, Lun Zhu, Yuhui Zhou, Edward Lau, James N. Smith, Anmol Arora, Charlotte Tulinius

**Affiliations:** ^1^ School of Clinical Medicine University of Cambridge Cambridge UK; ^2^ Lister Hospital, East and North Hertfordshire Trust Stevenage UK; ^3^ Department of Public Health and Primary Care University of Cambridge Cambridge UK

## Abstract

**Background:**

Clinical electives are a compulsory part of many medical courses, enabling students to gain exposure to foreign health systems. The COVID‐19 pandemic led to a surge in the development in virtual elective programmes, for which there had been a sparse evidence base. This is the first systematic review assessing the implementation, advantages and disadvantages of virtual elective programmes for medical students.

**Methods:**

The systematic review was conducted in accordance with PRISMA guidelines. Databases were searched, capturing results from the past 10 years for original evaluations of electives where medical students engaged in a fully virtual programme with another institution, with no restrictions on location. Descriptive, quantitative and qualitative data were extracted by two independent reviewers.

**Results:**

Fourteen articles were included for review. All the articles were published between 2020 and 2023. All studies were conducted in the United States of America. The average length of the virtual electives in the studies was 3 weeks, and a variety of teaching formats including virtual clinics, seminars and one‐on‐one meetings were implemented. Logistical considerations and challenges in delivering virtual electives included the variability in students' learning styles, reduction in patient interactions and technological challenges.

**Discussion:**

Most included studies derived that these virtual electives would play a role in the future, possibly replacing in‐person electives. Positive attributes of virtual electives included increasing diversity of social backgrounds, high student satisfaction and interest and reducing harms to the environment. However, further research is required to thoroughly evaluate the efficacy of virtual electives in medical education.

## 1 INTRODUCTION

A clinical elective in medical students' chosen specialty, institution and country is a compulsory part of the medical course in many medical schools across the globe. It allows medical students to experience clinical practice, improving their knowledge and insight into their chosen specialty. In addition, it provides opportunities for personal and academic growth.^1^ Studies suggest that as many as 50% of medical students in high income countries consider undertaking their elective abroad.^2^, ^3^ These, in particular, allow medical students to experience different health care systems and cultures, as well as influence future career decisions. However, alongside the benefits, this also calls into question the accessibility, cost and sustainability of such face‐to‐face electives. In particular, international medical electives (IMEs) could potentially pose the risks of infectious disease, personal violence and emotional distress.^4^

The COVID‐19 pandemic has caused massive disruption to health care systems and medical education, including in‐person electives and international placements. Travel restriction and suspension of many students' clinical activities have led to a significant decrease in the number of face‐to‐face IMEs.^5^ Indeed, in April 2020, 70% of medical schools in the USA received no visiting elective students.^6^ Similarly, the electives of 77.3% of British medical students, from a sample of 440 were postponed or cancelled, with comparable trends seen in two retrospective studies of German medical students.^6^, ^7^ As a result, virtual electives were introduced as an attractive alternative, with advantages such as the potential to improve students' access to a greater variety of elective opportunities and to be a more environmentally sustainable alternative even beyond the pandemic. However, despite the recent popularity of this elective format, whether results from virtual electives are comparable to those in person in a clinical setting and/or abroad remains unknown. There are limited data on medical students' experiences of electives in a virtual format, with the available data mostly coming from single‐centre studies.

The aim of this study was to investigate and analyse the potential advantages and disadvantages of the virtual elective format based on medical students' experiences. The primary research question was ‘What are the experiences of medical students who have undertaken virtual elective programmes?’

## 2 METHODS

This systematic review has been conducted in accordance with PRISMA guidelines (Data S1) and following a publicly available registered protocol.^8^

### 2.1 Search databases

Searches were run on 20 August 2023 on all databases. The search strategy (Appendix A) was intentionally broad, capturing a range of databases: MEDLINE, EMBASE, PSYCINFO, Web of Science and Eric. There were no restrictions on language. A 10‐year age limit was applied to searches, and papers ahead‐of‐print were eligible to be included. Reference lists of included papers were also screened, though no papers were selected through this manner.

### 2.2 Screening

Title and abstract screening was conducted in Rayyan.^9^ Each record was independently screened by two reviewers, each blinded to the decision of the other reviewer. Disagreements were resolved by a third independent reviewer where necessary. The following inclusion and exclusion criteria were applied during title and abstract screening as well as full‐text screening:

Inclusion criteria:

- Reports describing a short‐term placement forming part of a medical school curriculum, for which there is an opportunity to undertake it at a different institution to the medical school
- Reports describing electives that are wholly virtual or online
- Limit to post 2013 (past 10 years)

Exclusion criteria:

- Reviews, perspectives, opinions and letters that do not describe any specific implementation of a virtual elective project
- Implementation of projects for other professional groups, including qualified doctors, but not medical students
- Reports of electives requiring in‐person attendance.
- Online teaching modules that are do not involve live virtual interaction with a destination institution
- Projects that involve the use of recorded teaching material only
- Programmes describing virtual teaching within local curricula that does not include interaction with a destination institution separate to a local health care facility

Each record was screened in full‐text by two independent reviewers in a double‐blind fashion. Disagreements were resolved by involvement of a third independent reviewer where necessary.

### 2.3 Data extraction

A pilot data extraction was run in order to ensure that the protocol was robust at the time of extraction. During piloting, all authors had the opportunity to provide feedback on the data extraction protocol, though no changes were found to be needed. Data extraction for each article was performed by two reviewers independently using a predefined data collection matrix in the form of a Microsoft Excel spreadsheet. Data extractors were blinded to the identity and results of the other extractor for each article. Qualitative and quantitative data were extracted from included studies:

- Citation
- Date that the elective began
- Location of host institution
- Number of students enrolled
- Length of virtual elective programme
- Qualitative one‐to‐two line description of the project
- Quantitative metrics describing feedback from participants in the project (where available, qualitative feedback also to be recorded)
- Comments on the paper
- Any notable strengths or weaknesses identified by the reviewer

### 2.4 Synthesis

Each included study, therefore, had two sets of results. These were reviewed and reconciled by one author who produced the aggregate results table (Table 1) including both the qualitative and quantitative data collated together. If there was a lack of concordance between the two authors for a record, a third author may have become involved. This was not needed for any article. Data were collected in Microsoft Excel and visualised graphically in RStudio.^10^ Data were collated in tabular format for presentation in a manuscript with a list of documented virtual elective reports and their respective descriptions. A quality appraisal of included studies is included in Table B1.

**TABLE 1:** A summary of the included studies.

Index	Author	Year	Country	Study type	No of students	Response rate	Student grade	Length of project	Elective specialty	Summary of elective	Summary of survey
1	Bernstein et al.^11^	2021	USA	Quantitative survey	21	Post‐elective survey: 16/21 (76.2%)	Year 4	2 weeks	Head and Neck Surgery	Rotating schedule of teaching events on otolaryngology for medical students including journal club, discussion of cases, virtual grand rounds and virtual conferences.	How did they hear about the survey? Satisfaction with length of elective Satisfaction with schedule and organisation New level of interest about the residency programme
2	Dodelzon et al.^12^	2022	USA	Quantitative survey	3	Pre‐elective survey: 3/3 (100%) Post‐elective survey: 3/3 (100%)	Year 4	4 weeks	Radiology	Variety of virtual teaching sessions based on radiology were delivered and pre‐ and post‐programme surveys were completed by all 3 students.	Course content Interest in specialty Insight to specialty
3	Gunther et al.^13^	2020	USA	Quantitative survey	27	Pre‐elective survey 22/27 (81.5%) Post‐elective survey 20/27 (74.1%)	Year 4	1 week	Radiation Oncology	Virtual elective in radiation oncology to replace in‐person elective. The virtual elective used a lecture series and interactive virtual events. Pre‐ and post‐elective survey carried out.	Pre‐elective Benefits and value of participation Post‐elective Benefits and value of participation
4	Haws et al.^14^	2020	USA	Quantitative survey	24	Post‐elective survey: 23/24 (95.8)	Year 4	2 weeks	Trauma and Orthopaedic Surgery	Virtual orthopaedic elective was offered to 4th year medical students. Variety of teaching sessions were held on zoom and a post‐programme survey was used.	Satisfaction with elective Influence of elective on perspectives on residency Thoughts on potential substitute for an in‐person programme Perceptions on interactions with faculty members
5	Hoffman et al.^15^	2020	USA	Quantitative survey	27	Post‐elective survey: 20/27 (74.1%)	Year 4	1 week	Neurosurgery	Virtual neurosurgical elective for 3rd and 4th year medical students utilising case discussions, interactive review sessions via Zoom. Post course survey was carried out using 3‐point Likert scale.	Learning and satisfaction with different sessions
6	Koch et al.^16^	2020	USA	Quantitative & qualitative survey	9	Post‐elective survey: 9/9 (100%)	Year 4	4 weeks	Pathology	Virtual pathology elective designed to mimic in person elective with lectures, small group teaching. Post elective survey and in‐person feedback was conducted.	Ratings of faculty members and individual course components How to improve the course What helps the learning process
7	Lee et al.^17^	2020	USA	Quantitative survey	49	Post‐elective survey: 32/49 (65.3%)	Year 4	4 weeks	Otolaryngology	Virtual otolaryngology medical elective. Department grand rounds, teaching conferences, consult sessions, resident socials, virtual meet and greets. Hosted through Zoom.	Usefulness of each component Success of elective in meeting objectives Success of elective in providing opportunities
8	Villa et al.^18^	2021	USA	Quantitative & qualitative survey	26	Pre‐knowledge test: 26/26 (100%) Post‐knowledge test: 26/26 (100%) Post‐ elective survey: 25/26 (96.2%)	Year 4	2 weeks	Emergency Medicine	Virtual emergency medicine elective. Didactic teaching sessions and case reviews. Evaluated with pre‐ and post‐programme tests and evaluative surveys.	Quality of learning Variety of learning topics Adequate focus on social determinants of health Impact of programme on residency choices Opinion on whether the programme should be repeated
9	Belfi et al.^19^	2020	USA	Quantitative & qualitative survey	26	Post‐elective survey: 26/26 (100%)	Year 4	2 weeks	Radiology	Virtual 2‐week introductory radiology elective. Daily assignments were developed for students including online learning modules. There was a radiology resident conference, lectures, virtual readout sessions and an assessment at the end.	Educational value of survey Level of engagement Impression of course Delivery of the course
10	Mason et al.^20^	2020	USA	Quantitative survey	31	Post‐elective survey 18/18 (100%)	Year 4	4 weeks	Trauma and Orthopaedic Surgery	Virtual orthopaedic surgery clerkship for which 31 fourth‐year medical students participated. Each clerkship included 8 two‐hour education sessions.	Strengths and weaknesses of programme Opinions on whether virtual elective format should be repeated in the future
11	Reghunathan et al.^21^	2020	USA	Quantitative survey	26	Pre‐elective survey 25/26 (96.2%) Post‐elective survey 22/26 (84.6%)	Year 4	2 weeks	Plastic and Reconstructive Surgery	Virtual plastic surgery elective comprising case reviews, inpatient rounds, virtual suture coaching, mentorship meetings and research meetings.	Perceived objectives of programme and how well these objectives were met
12	Rydberg et al.^22^	2021	USA	Quantitative survey	87	Post‐elective survey: 61/87 (70.1%)	Year 1	8 weeks	Physical Medicine and Rehabilitation	Virtual physical medicine and rehabilitation elective.	Content of elective programme Insight provided by the elective to the specialty
13	Sandhu et al.^23^	2020	USA	Quantitative & qualitative survey	26	Post‐elective survey: 26/26 (100%)	Clinical year 1: 21 (80.8%) Clinical year 2: 5 (19.2%)	4 weeks	Oncology	Virtual oncology elective including a virtual clinic, lectures and other educational sessions.	Experience of each component of the elective Insight provided by the elective to the specialty Interest in specialty after elective
14	Tucker et al.^24^	2020	USA	Quantitative & qualitative survey	12	Pre‐elective survey 8/12 (75%) Post‐elective survey 5/212 (41.7%)	Not stated	2 weeks	Plastic and Reconstructive Surgery	Virtual plastic surgery elective, designed to mimic the exposure and educational opportunities afforded by a face‐to‐face elective.	Pre‐elective questionnaire ‐Presence of a plastic surgery placement in their host medical curriculum Post‐elective questionnaire ‐Enjoyment of program ‐Opinion on whether the virtual format should be repeated

## 3 RESULTS

### 3.1 Study characteristics

**FIGURE 1:**
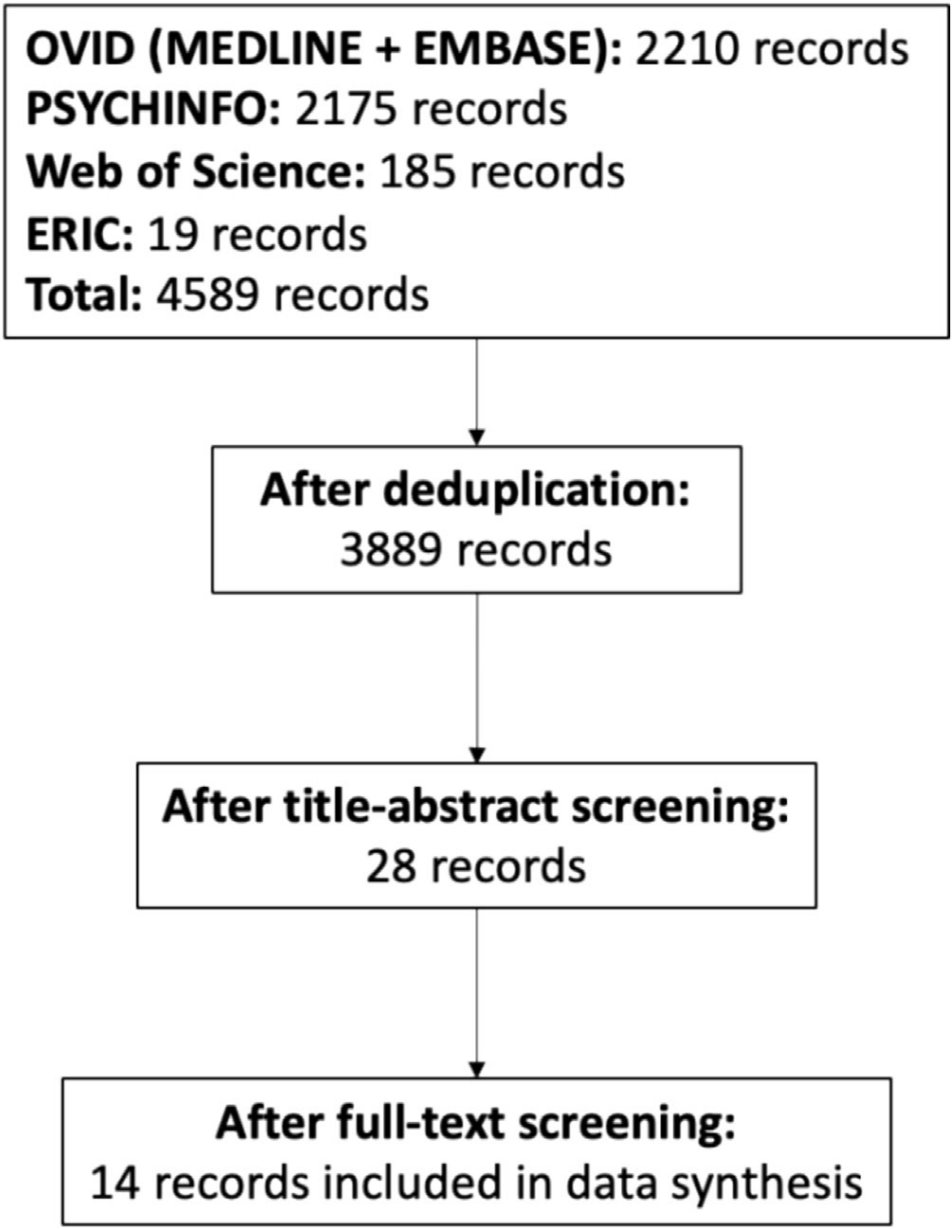
PRISMA diagram showing the inclusion of 14 unique studies after full‐text screening, from a total of 3,889 unique records identified by searches. Reasons for exclusion were not routinely recorded.

After removing duplicates, the initial search produced 3,889 records. Screening of the aforementioned articles produced 28 records for full text review. Of these, 14 records were included in this systematic review after applying the inclusion and exclusion criteria (Figure 1).

The 14 articles that were included in this systematic review had publication dates between 2020 and 2023. They were all descriptive in their design. All studies utilised a variety of mixed methods, quantitative and qualitative approaches in their approaches. Four studies carried out pre‐elective surveys. All studies employed a post‐elective survey of the medical students that participated. Three studies also surveyed faculty members. Although response rates varied between studies, the majority of participants in each elective programme completed a post‐elective survey. The primary centre for most studies was in the USA.

Length of virtual electives varied between 1 and 8 weeks, with a mean of 3 weeks. The virtual electives used a variety of virtual teaching sessions including grand rounds, consultations, lectures and 1:1 feedback and teaching sessions, though the data were not consistently reported in a manner that allows specific quantification.

**FIGURE 2:**
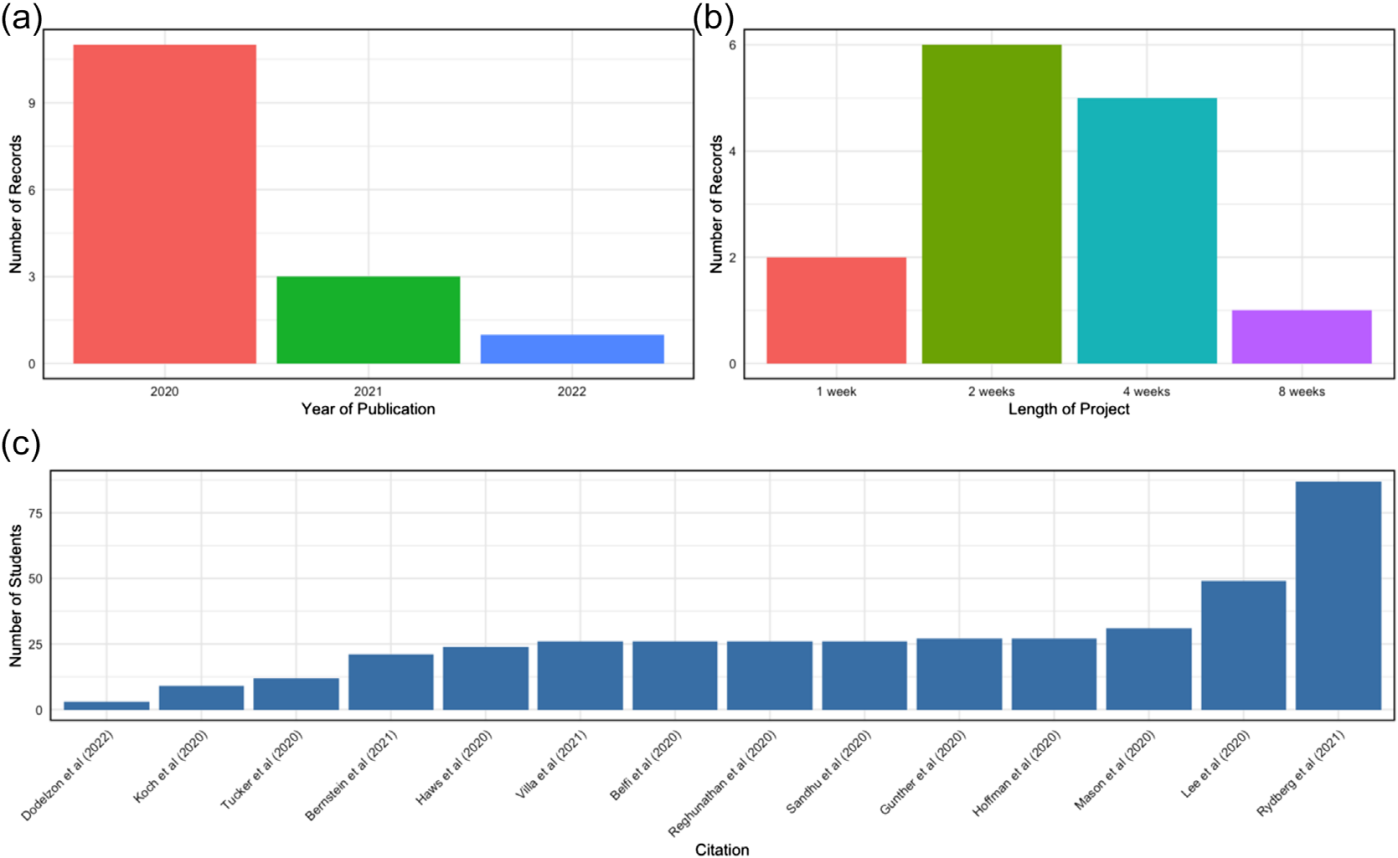
Graphical representation of the characteristics of included studies. (a) Year of publication of included studies. (b) Length of virtual elective programme. (c) Number of students who undertook each virtual elective programme.

A summary of the included studies is included in Table 1 and illustrated in Figure 2. Please see Appendix B for a table detailing the data extracted from the included studies.

### 3.2 Student satisfaction and interest

Student satisfaction—defined broadly including whether students would recommend to a friend and if they would take part in the elective again—was high amongst all the studies included. Students gave positive feedback on the level of engagement that virtual electives enabled. In particular, students highly valued time spent in virtual clinics, meeting faculty members and the insights the virtual electives gave them into residency. Whether the electives influenced interest in the specialty was not uniformly asked but where reported results were positive.^23^

### 3.3 Virtual versus in‐person elective programmes

Preferences for the format of electives varied amongst the studies. Some students enjoyed the reduced pressure and increased flexibility associated with virtual electives,^18^ while others maintained a preference for traditional in‐person elective format,^21^ though not every study included in our review asked students these particular questions. Villa et al.^18^ demonstrated that 95% of the medical students surveyed agreed that the virtual elective should be continued in the future. However, another showed that only 20% of students preferred a virtual elective over an in‐person one.^21^ Conversely, Koch et al.^16^ showed the opposite effect, where 100% of students preferred the virtual format.

### 3.4 Diversity and inclusivity

The authors of a number of studies indicated the potential of virtual electives to increase diversity within medicine by increasing accessibility,^11^, ^14^, ^16^, ^17^, ^18^ particularly for underrepresented minority students. One study only recruited students from backgrounds that are underrepresented within medicine.^12^ One of the main ways that virtual electives reduce barriers to increased diversity within medicine is by reducing the costs, which accompany traditional in‐person electives.^16^ Other barriers to diversity within medicine when considering electives might include travel restrictions due to mobility or commitments such as caring responsibilities.

### 3.5 Logistical considerations

The virtual elective programmes that were studied were instituted as a direct consequence of the public health measures that were necessitated by the COVID‐19 pandemic. The institutions that acted as the centres for these studies would not have been able to host an elective programme if it was not virtual. As a result, virtual electives were an attractive and important alternative for medical students and institutions of medical education. This evidences the logistical advantages of virtual elective programmes, in particular their adaptability and flexibility.

### 3.6 Challenges

Common challenges included the absence of direct patient interactions, limited 1:1 time and technological difficulties during virtual surgical procedures. In addition, some students had a distinct preference for in‐person lectures over virtual lectures, highlighting the interpersonal variability between learning styles.

## 4 DISCUSSION

### 4.1 Summary of key findings

This is the first systematic review evaluating virtual electives and their role within medical education. The majority of current evidence has arisen as a consequence of the COVID‐19 pandemic. The findings of this review demonstrate a consistently high level of student satisfaction with virtual elective programmes, consisting of grand rounds, lectures, group teaching, inpatient rounds and clinics. In addition, the students' interest levels in the specialty that the elective was undertaken in was either maintained or increased. The majority of students predicted that virtual electives would have a part to play in medical education. These findings suggest that virtual electives were positively received by medical students and can act as an effective and engaging alternative to traditional electives in the future.

The variability in student preference noted in the results suggests that there is no clear favourite amongst students between virtual and in‐person electives. Possible reasons for this may include differences in learning styles between individual students or differences between the composition of the virtual electives. In addition, some specialties lend themselves more kindly to a virtual elective than others. For example, Reghunathan et al.^21^ demonstrated that only 20% of students displayed a preference for virtual electives. However, this study investigated a virtual elective in plastic surgery—a speciality where a virtual elective may find it more difficult to mimic an in‐person elective than, for example, a virtual elective in radiology.

Students repeatedly expressed high levels of satisfaction with scenarios involving small groups, one‐to‐one sessions and networking with faculty members. This suggests that designing a successful virtual elective programme requires intentional design to promote human interaction and connection.

Logistical advantages, such as reduced cost and increased accessibility, highlight important positive factors associated with virtual electives. However, challenges still remain including reduced time spent with patients and one‐on‐one with physicians. The small cohort sizes in the study and the value that students derive from small group sessions may reduce the scalability and therefore accessibility of virtual electives.

### 4.2 Comparison to existing evidence

While there are undoubtedly many benefits to virtual electives, one has to consider what is missed in comparison to IMEs in a global health context. There are certainly benefits to working with people from culturally, linguistically and socioeconomically diverse backgrounds and a richness in experience, which may be omitted during a virtual elective.^25^ It is also important to consider that virtual electives are able to provide differing levels of quality to students depending on the specialty chosen and the educational environment the elective is set in, with large differences in radiology compared with surgery for example. The benefits of ‘wellness’ electives to help students relieve stress during medical school have also been shown to provide students with ways of better managing issues in their personal and professional lives, and it is known that a focus on wellness is part of IMEs, with students opting to take cultural side trips and other adventures.^4^, ^26^ That said, these ‘wellness’ benefits need not necessarily be lost with virtual electives.

While there are financial and environmental advantages to virtual electives, the option for students to undertake electives within their home country—‘domestic’ electives—should be included in evaluations of virtual electives. Domestic electives may have a similar financial and environmental advantage to virtual electives while also enabling the richness of experience that being physically present in a health care environment provides. It is notable that in some systems of medical education, domestic electives are more commonly undertaken. Ali et al.^1^ found that the majority of medical students in Pakistan chose to undertake their electives domestically. This is in part due to the financial burden of travelling overseas, the ease of finding an elective locally and the restrictions overseas electives put on their academic careers due to clashes between overseas electives and Pakistan's academic curriculum at medical school. They did note however that students can benefit more from IMEs due to the diversity of medical conditions seen and the opportunity to be physically present in a new learning environment.

Carrying out virtual electives may also reduce the ability for students to contribute to ‘globalisation’. The term globalisation is often misused to explain many natural and human induced changes, but globalisation can be defined as processes that change the way people interact across physical, cultural and other forms of boundaries, resulting in a redefining of human society.^26^ A factor that affects delivery of health care across countries, globalisation requirements are often met by the opportunities presented to students during IMEs. Students also reported meaningful outcomes in medical knowledge, skills, health care organisation and personal growth. It appears, however, that these experiences often lack mutuality, a phenomenon that aims to mitigate power differentials through equity, autonomy, solidarity and participation.^27^ There is a lack of research into the benefits that the recipient community receives when students are placed abroad suggesting the benefits of IMEs could be one sided.^28^

In addition, IMEs can often cause a variety of issues for students who undertake them. For example, a separate study also found there were many cultural, political, physical and moral problems encountered by students during IMEs.^3^ One female student carrying out an IME in Egypt felt that women had to present themselves as timid and dress conservatively, while another faced a moral dilemma related to treating patients and practising outside of their competency as a medical student and a third was practising in a country experiencing civil unrest. Lumb and Murdoch‐Eaton^29^ discuss the fact that electives are a less monitored section of the medical curriculum, but it is likely that the use of virtual electives would allow monitoring to be carried out more easily, since they could be designed by medical schools with specific objectives. Conversely, the authors also discuss how IMEs provide students with transferable skills through working with people from a variety of backgrounds socioeconomically and culturally.

Furthermore, it is possible that partnerships pose a risk of scientific colonisation. For example, wealthier countries may impose their expectations of curriculum on poorer host countries to benefit visiting students, without consideration of whether this is reflective of the health and educational needs of that country. Western institutions could display a ‘saviour mentality’, whereby they feel their students represent a higher standard of practice and that their role is to educate, rather than learn from, their host. However, the drive for these partnerships can come from both low and high resource countries. Developing countries may feel an obligation to partner with institutions in high income countries even when unreasonable expectations are set, to gain access to academic publication opportunities that are otherwise inaccessible to them. Either way, electives that become transactional may be detrimental to the development of a sustainable and meaningful partnership.^27^ In contrast, electives that are genuinely equitable and reciprocal can help reduce any detrimental effects, preventing the perpetuation of ongoing coloniality and international power imbalances. Both IMEs and virtual electives have the potential to be genuinely equitable, and steer clear of coloniality if a conscious effort is made by both parties. However, it could be argued that virtual electives could provide a faster, simpler path in this direction due to the lower financial costs and greater accessibility associated with the lack of travel that access to relatively cheap, virtual programmes could provide.^30^ This, of course, will still require cooperation between countries to ensure an equitable relationship when developing a virtual partnership.

### 4.3 Implications of findings

The results of this systematic review support the more widespread roll out of virtual electives for medical students. Virtual electives increase accessibility and reduce cost as well as reducing the carbon footprint—an important consideration in an increasingly environmentally conscious world. It is important that a virtual elective meets the same needs that an in‐person elective would, for example, developing international relationships and knowledge while adding value to their host institution. Currently, virtual electives require advanced technology mostly available in developed countries, but in future, there is scope for virtual elective programmes to be utilised in developing countries to increase accessibility for students in these areas.

### 4.4 Limitations

The collective findings of the studies provide valuable insights into virtual electives and their role within medical education. Despite this, it is important to acknowledge the limitations in these studies. A recurrent limitation across the included studies is the small sample sizes. This limitation is compounded by the inherent biases present including self‐selection bias—as the most enthusiastic students are more likely to sign up to such programmes—and recency bias. Furthermore, students generally undertook electives at centres where they were considering applying to residency positions shortly afterwards. This may have resulted in latent positive skewing of the feedback in an attempt to please future interview panels and colleagues.

It is important to distinguish between virtual elective programmes and virtual teaching programmes. Different institutions may use different terminology for ‘electives’, and so we sought to broaden our scope as much as possible to ensure that relevant results were included. Results where students engaged in a virtual programme of teaching at their own institution were not considered ‘virtual electives’, even if they were designed to replace electives in medical school curricula. To fulfil the inclusion/exclusion criteria for the review, the content of the teaching programme must have been different from the student's medical school. The teaching programme must have also been of a short duration. While we did not set a strict criteria for what defines a short programme, we felt that this should encompass ‘weeks to months’, rather than a year abroad. When conducting screening. we erred on the side of the inclusion if there was any doubt as to whether a programme was of a suitable duration.

Another important limitation is the diversity in the elective duration, content and methodologies across studies. As a result, it is challenging to make direct comparisons between studies. The brief duration of the virtual electives also makes comparison to in‐person electives difficult as such programmes generally last for over 4 weeks. In addition, none of the studies provided a breakdown of costs associated with their virtual elective. Therefore, it was also not possible to compare this aspect of virtual electives to in‐person electives.

The majority of the studies provided feedback from students who carried out virtual electives during the COVID‐19 pandemic, when IMEs were often not feasible. Therefore, the medical students did not have a direct comparison or reference point when completing the feedback. Many of the medical students would not have had an experience of a traditional in‐person elective as a result of the pandemic. Furthermore, the impact of reduced patient interactions on the future careers of the medical students was not possible to evaluate from the studies included.

Finally, the USA focus of these studies limits the generalisability of conclusions to other countries. Differences in educational systems between nations is large, particularly in medical education, reducing the applicability and transferability of these findings to other nations. In addition, the USA focus suggests that there may be a bias towards virtual electives occurring predominantly in high income rather than low and medium income countries.

### 4.5 Directions of future research

Future research into virtual medical electives should expand current understanding of the advantages and disadvantages of virtual electives including when they may be used most effectively within medical education. Expanding the geographical range of this research beyond the USA, to include international perspectives, is critical to capturing a larger dataset, exploring the availability of virtual electives in low and middle income countries and providing an insight into the nuances of virtual electives in a variety of educational and cultural contexts. There is a need to hear the voices of the other stakeholders involved in medical electives, including health professionals and patients, and in particular lower and middle income countries.

The reduced carbon emissions produced through virtual electives in comparison to international electives is also an area that has been overlooked, mostly due to the lack of current literature on this topic. Future studies should factor this benefit in when comparing elective types because it will likely be a significant advantage of virtual electives as the climate crisis escalates and societies respond to this. However, reducing carbon emissions could come at a price, particularly to lower resource host countries as they may gain from developing electives to host foreign students. Therefore, it would be important to incorporate this factor by listening to host countries.

This review has demonstrated a lack of standardisation amongst studies investigating virtual medical electives. Standardised studies could enable better comparisons to be made to traditional in‐person electives and also between virtual electives.

One of the findings of the systematic review highlighted that specific components of the virtual electives can contribute to high student satisfaction. Therefore, future research should explore the preferences and experiences of students further to inform the development of more effective virtual elective models.

Finally, the studies used mainly a post‐elective questionnaire to collect data. Integration of other methodologies such as focus groups, in‐depth interviews and quantitative analyses can add to the current literature within the field.

## AUTHOR CONTRIBUTIONS

**Luke McCarron:** Investigation; data curation; writing—original draft; visualization. **Nihal Sogandji:** Investigation; data curation; writing—original draft. **Luke Coakham:** Investigation; data curation; writing—review and editing. **Lun Zhu:** Investigation; data curation; writing—review and editing. **Yuhui Zhou:** Methodology; data curation; investigation; writing—review and editing. **Edward Lau:** Methodology; data curation; investigation; conceptualization; supervision; writing—review and editing. **James Smith N:** Conceptualization; methodology; investigation; data curation; writing—review and editing; supervision. **Anmol Arora:** Conceptualization; methodology; investigation; writing—review and editing; data curation; supervision; visualization; project administration. **Charlotte Tulinius:** Conceptualization; methodology; investigation; writing—review and editing; project administration; supervision.

## CONFLICT OF INTEREST STATEMENT

The authors have no conflict of interest to disclose.

## ETHICS STATEMENT

The authors have no ethical statement to declare.

## Supporting information


**Data S1.** Supporting Information.

## Data Availability

The data that support the findings of this study are available in the supporting information of this article.

## APPENDIX A

### A.1 Search Query Strings

ERIC:

1. ((virtual or remote or online or distance) AND (placement* or elective* or attachment* or rotation* or intern* or clerkship* or exchange* or program* or experience*)) 110
  OR
2. (‘Collaborative online international learning’) 5251
3. 1 OR 21105348
4. ((student or trainee or undergraduate*) AND (doctor* or medic* or clinical)) 2850
5. 3 AND 4 19

(((virtual or remote or online or distance) AND (placement* or elective* or attachment* or rotation* or intern* or clerkship* or exchange* or program* or experience*)) OR (‘Collaborative online international learning’)) AND ((student or trainee or undergraduate*) AND (doctor* or medic* or clinical))

PSYCINFO:

1. TI (((student OR trainee OR undergraduate*) AND (doctor* OR medic* OR clinical))) OR AB (((student OR trainee OR undergraduate*) AND (doctor* OR medic* OR clinical))) OR KW (((student OR trainee OR undergraduate*) AND (doctor* OR medic* OR clinical))) 68527
2. MA MEDICAL STUDENT 6467
3. TI (((virtual or remote or online or distance) AND (placement* or elective* or attachment* or rotation* or intern* or clerkship* or exchange* or program* or experience*))) OR AB (((virtual or remote or online or distance) AND (placement* or elective* or attachment* or rotation* or intern* or clerkship* or exchange* or program* or experience*))) OR KW (((virtual or remote or online or distance) AND (placement* or elective* or attachment* or rotation* or intern* or clerkship* or exchange* or program* or experience*))) OR MA (((virtual or remote or online or distance) AND (placement* or elective* or attachment* or rotation* or intern* or clerkship* or exchange* or program* or experience*))) 85334
4. TI Collaborative online international learning OR AB Collaborative online international learning OR KW Collaborative online international learning 9
5. S1 OR S2 69109
6. S3 OR S4 85334
7. (S3 OR S4) AND (S5 AND S6) 2904
8. (S3 OR S4) AND (S5 AND S6) Limiters ‐ Publication Year: 2013–2023 2175

WEB OF SCIENCE:

1. TS = (((student or trainee or undergraduate*) NEAR/0 (doctor* or medic* or clinical))) 70608
2. TS = (((virtual or remote or online or distance) NEAR/0 (placement* or elective* or attachment* or rotation* or intern* or clerkship* or exchange* or program* or experience*))) 14,210
3. TS = (‘Collaborative online international learning’) 76
4. 2 OR 314210
5. 1 AND 4212
6. #1 AND #4 and 2023 or 2022 or 2021 or 2020 or 2019 or 2018 or 2017 or 2016 or 2015 or 2014 or 2013 (Publication Years) 185

OVID:

1. ((student or trainee or undergraduate*) adj (doctor* or medic* or clinical)).ti,ab, kw,kf.
2. exp Education, Medical, Undergraduate/or exp Students, Medical/
3. 1 or 2
4. ((virtual or remote or online or distance) adj3 (placement* or elective* or attachment* or rotation* or intern* or clerkship* or exchange* or program* or experience*)).ti,ab, kw, kf.
5. Collaborative online international learning.ti,ab,kw,kf.
6. 4 or 5
7. 3 and 6
8. limit 7 to vr = ‘2013–Current’

## APPENDIX B

**TABLE B1:** Data collected extracted from the included studies.

Author	Quantitative	Comments	Strengths and weaknesses
Bernstein et al.^11^	16 students filled in a post survey questionnaire. 73% of respondents said the duration was appropriate, 13% wanted longer and 13% shorter. 73% reported being highly satisfied with the programme. 100% reported continued interest in applying to otolaryngology residency programmes, 87% reported a greater level of interest in the programme than before participating. 100% reported feeling extremely comfortable with topics related to the residency programme.	The authors state that virtual internships cannot replace in person but that they are convenient and effective. The authors also noted that students would value more opportunities to demonstrate their worth to the department.	Strengths: Mixed methods study; some insight into what students would value in future programmes. Weaknesses: low number of participants; potential for self‐selection bias as only those interested would apply and reply to the survey; potential for recency bias as survey was given shortly after completing the programme; finally, participants may have felt compelled to rate the virtual elective more highly than they believed.
Dodelzon et al.^12^	All three students agreed they would recommend the course to other medical students and that it gave them insight into a career of a radiologist. Most aspects of the course were rated as high or very high quality by participants.	Recruitment was targeted at underrepresented minority medical students	Strengths: small study showing how virtual electives could be used to increase diversity in medicine. Weaknesses: small sample size.
Gunther et al.^13^	22 students (81%) completed the pre‐elective survey. 20 (74%) completed the post‐elective survey. In the post‐elective survey, 80% (16) of students preferred a full onsite away elective whereas 20% (4) preferred a virtual elective. Interactions with faculty residents and networking were rated highly important on both pre‐ and post‐elective survey.		Weaknesses: small sample size; potential for response bias as participants may have been positively biassed in their responses
Haws et al.^14^	96% (23) students completed the survey. 87% felt very satisfied, 9% somewhat. 35% found very useful substitution for in‐person, 61% somewhat. 96% strongly satisfied with interactions with residents. Faculty members: 18/22 completed survey. Mixed responses from faculty members as to how useful the virtual programme was.	Feedback completed by faculty members in addition to students.	Strengths: High retention rate for survey completion (23/24); Weaknesses: small sample size; performed at a single institution; potential for response bias as despite surveys being anonymous the students were in the middle of their application cycle to residency programmes at the host institution.
Hoffman et al.^15^	74% (20/27) responded to the survey. 100% of respondents would recommend it to other students. Top 3 words used to describe the elective were ‘interactive’ ‘engaging’ ‘educational’. Low pressure and welcoming environment. 6/8 faculty responded, 33% stated candidate insight would ‘definitely’ influence residency ranking decisions and 67% stated ‘maybe’.	Feedback completed by faculty members in addition to students.	Strengths: six international students enrolled. Weaknesses: X (formerly Twitter) was used as the primary recruitment platform; short duration of programme.
Koch et al.^16^	100% (9) respondents thought the rotation was extremely beneficial and that they were ‘likely’ or ‘very likely’ to participate in future sessions. The participating trainees preferred the virtual rotation to in‐person rotations.	Feedback completed by faculty members in addition to students. Programme designed to mimic in person rotation.	Strengths: survey completed by all 9 students. Weaknesses: small sample size.
Lee et al.^17^	32 participants completed the post‐programme survey (65% response rate). Six students (19%) reported not having a home institution otolaryngology programme. The activities rated as being most useful were the virtual ‘on‐call’ consult sessions, resident socials, and faculty meet‐and‐greets.	The authors noted that ‘A successful virtual sub‐internship holds abundant potential for future medical students applying into surgical subspecialties. Even once the pandemic subsides, in‐person away rotations incur significant financial and logistical costs’	Strengths: aimed to recreate typical visiting student rotations. Weaknesses: no mention of direct patient interactions.
Villa et al.^18^	25/26 (96%) students completed the end‐of‐rotation evaluative survey. Of all respondents, 95% ‘strongly agreed’ or ‘agreed’ that the rotation should be repeated in the future. Statistically significant improvement in pre/post‐tests (71.7% vs. 76.3%). 75% of surveys completed, 89% of respondents strongly agreed topics were important to ED, 66% more confident after completing modules	Authors indicated that they would likely offer the virtual internship again as it is significantly cheaper for students and therefore can cater to medical students from communities which are traditionally underrepresented in medicine	
Belfi et al.^19^	Pre‐ and post‐elective assessment showed improvement from an average score of 34/55 to 49/55. 100% response rate for post course survey, 21/26 reported that they have not taken a similar virtual course before, all reported that they would recommend this elective to other students. Overall, all the students were more comfortable looking at imaging studies, helping them to prepare for clinical rotations and future careers in radiology. The virtual medical student lectures received an overall score of 4.92/5 (5 = very high quality).	Students reported that they thought the modules were interactive, informative and enjoyable.	Strengths: 100% response rate, Weaknesses: small sample size
Mason et al.^20^	Of the visiting students (18), 10 rated the experience as outstanding, and eight rated it as excellent. Thirteen would highly recommend the programme. Sixteen students believe virtual clerkships would have a role moving forward and two did not.		Weaknesses: relatively small amount of time spent doing formal elective programmes ‐ 16 hours in total; small number of respondents to post‐elective survey
Reghunathan et al.^21^	100% of respondents said that they would participate in the virtual elective again. However only 20% of respondents said they would choose a virtual elective over an in‐person elective.		Strengths: Compared to face‐to‐face elective more time with intern, reduced cost, flexible schedule; individualised goal setting.
Rydberg et al.^22^	Respondents almost unanimously ‘agreed’ or ‘strongly agreed’ that ‘My knowledge of the field of PM&R and scope of practice of PM&R physicians has improved by participation in the Virtual PM&R Experience’ (4.79 on a 1–5 Likert scale) and that ‘This experience gave me a realistic preview of my field of interest’ (4.73 on a 1–5 Likert scale). Technology and communication, including access to the online materials and small‐group discussions, were highly rated overall by students (4.32 on a 1–5 Likert scale). One hundred percent of respondents would either recommend or strongly recommend the Virtual PM&R Experience to other medical students.		
Sandhu et al.^23^	Sixteen (61.5%) students agreed or strongly agreed that the elective helped inform their decision to choose a specialty; 6 (23.1%) remained neutral, and 4 (15.3%) disagreed or strongly disagreed.		Weakness: small sample size with only four visiting students; no student feedback on how this virtual programme compares to in‐person etc
Tucker et al.^24^	Three students form the elective went on to apply for in person rotations. On average, students spent 55.7 hours actively engaging during the 2‐week rotation. The pre‐elective survey was completed by 8 of 12 students (75%), and the post‐elective survey by 5 of 12 students (42%). Overall, students enjoyed the rotation and found it useful and informative. When asked if the virtual option should be kept for next year, 60% (3 of 5) answered ‘yes’ and 40% (2 of 5) answered ‘maybe’. Fifty percent (4 of 8) of students reported planning to do another virtual rotation. The elective increased students' familiarity with multiple aspects of the residency programme and lifestyle.		Strengths: students got to experience virtual operations
